# West Nile Virus Infection in Occupational Settings—A Systematic Review

**DOI:** 10.3390/pathogens13020157

**Published:** 2024-02-09

**Authors:** Amienwanlen E. Odigie, Angela Stufano, Valentina Schino, Aya Attia Koraney Zarea, Linda A. Ndiana, Daniela Mrenoshki, Iniobong C. I. Ugochukwu, Piero Lovreglio, Grazia Greco, Annamaria Pratelli, Michele Camero, Maria Tempesta

**Affiliations:** 1 Department of Veterinary Medicine, University of Bari Aldo Moro, SP 62 Casamassima km 3, 70010 Valenzano, Italy; amienwanlen.odigie@uniba.it (A.E.O.); vetaya.nrc@gmail.com (A.A.K.Z.); laukagwu@gmail.com (L.A.N.); danimrenoski@yahoo.it (D.M.); iniobong.ugochukwu@unn.edu.ng (I.C.I.U.); grazia.greco@uniba.it (G.G.); annamaria.pratelli@uniba.it (A.P.); michele.camero@uniba.it (M.C.); maria.tempesta@uniba.it (M.T.); 2 Department of Veterinary Public Health, University of Benin, Benin City 301154, Nigeria; 3 Interdisciplinary Department of Medicine-Section of Occupational Medicine, University of Bari, 70124 Bari, Italy; valentina.schino@uniba.it (V.S.); piero.lovreglio@uniba.it (P.L.); 4 Department of Microbiology and Immunology, Veterinary Research Institute, National Research Centre, Dokki 12622, Egypt; 5 Department of Veterinary Microbiology, College of Veterinary Medicine, Michael Okpara University of Agriculture, Umudike 440101, Nigeria; 6 Department of Veterinary Pathology and Microbiology, University of Nigeria, Nsukka 410001, Nigeria

**Keywords:** West Nile virus, mosquito-borne disease, workers, risk, occupational exposure

## Abstract

Background: West Nile virus (WNV) is an emerging mosquito-borne neurotropic virus, belonging to the *Flaviviridae* family and the *Orthoflavivirus* genus. The effective control of WNV requires a targeted preventive strategy that also needs the identification of the higher-risk populations. Hence, this study focused on a systematic literature review of WNV-acquired infection in work-related settings and the assessment of the exposure risks among different occupational categories. Methods: A comprehensive search was conducted to identify studies until September 2023 in multiple databases such as PubMed/MEDLINE, SCOPUS and Web of Science, according to the PRISMA 2020 statement. Risk of bias of collected papers was assessed by the ROB tool of the National Toxicology Program’s Office of Health Assessment and Translation handbook. Results: A total of 21 studies were included in the systematic review, out of which seventeen were observational studies and four were case reports. Workers identified as at higher risk for WNV infection were military workers, veterinarians, agricultural workers, farmers, and laboratory workers with contact with infected fluids or aerosols. Conclusions: The identification of higher-risk workers could facilitate active surveillance by occupational physicians, which could improve our understanding of the epidemiology of WNV and, in addition, could help tailor appropriate preventive recommendations, reducing the overall burden of disease in high-risk areas.

## 1. Introduction

West Nile virus (WNV) is a mosquito-borne neurotropic virus, belonging to the *Flaviviridae* family and the *Orthoflavivirus* genus, and is a member of the Japanese encephalitis serocomplex [1]. It is an enveloped, single-stranded positive-sense spherical RNA virus of approximately 50 nm in diameter. The three viral structural proteins, capsid (C), membrane (prM/M) and envelope (E), are encoded within the 5′ portion of the ORF, while 7 nonstructural proteins (NS1, NS2A, NS2B, NS3, NS4A, NS4B and NS5) are encoded within the 3′ portion [2]. WNV can be divided genetically into nine lineages, but only lineage 1 (WNV-1), identified in North America, North Africa, Europe and Australia, and lineage 2 (WNV-2), endemic in South Africa and Madagascar, can cause human disease [3].

In the recent years, WNV spread has become an emerging public health issue, with the onset of outbreaks also in a certain number of European countries including Italy, Hungary, Romania and Greece [4]. Since the beginning of the 2023 transmission season and as of December 2023, European countries have reported 707 human cases of WNV infection in Italy, Greece, Romania, France, Hungary, Spain, Germany, Croatia and Cyprus [3]. This spreading of WNV infections in Europe has been associated to the climate characteristics reported during the last period and to the introduction of new lineage 1 WNV genetic variants [3].

The main transmission route of WNV to humans is through infected mosquito bites during feeding, with *Culex* species by far the most common vector in the US, Europe, Australia and South Africa. In the meantime, there were up to 45 species and eight genera of mosquitoes implicated in the transmission of WNV in the US between 2004 and 2008 [5]. WNV is amplified only in mosquitoes and avian species, with migratory birds playing an important role in spreading the virus [6]. Infected birds develop transient viremia levels high enough to infect feeding mosquitoes, sustaining the transmission cycle. Humans and horses are incidentally infected by WNV through mosquito bites, although they are regarded as dead-end hosts as they neither participate in the WNV lifecycle nor develop sufficient viremia to infect mosquito vectors [7]. Outbreaks in wild birds and horses usually occur before the report of human infection and are considered a reliable indicator for a possible human WNV epidemic season [8,9]. Other possible routes of transmission have occasionally been described in humans, such as organ transplantation, blood product transfusions, trans-placental and breast-feeding transmission, and percutaneous exposure [10,11]. Moreover, experimental evidence of aerosol transmission has been reported in mice [12].

Clinically, there are three possible courses of human WNV infection, linked to the host’s immunity and/or virus strain. About 80% of human infections are asymptomatic, while the West Nile fever (WNF) shows self-limiting flu-like symptoms. The less frequent course is the WNV neuroinvasive disease (WNND), comprising approximately 0.04% to 0.07%, characterized by meningitis, encephalitis, or acute paralysis, with a fatality rate of 10% among the elderly [13]. Both in WNF and WNND, long-term sequelae of weakness, fatigue and cognitive deficits have been observed for up to 18 months after the disease onset. Currently, 85 years since it was first identified, there is no licensed vaccine to control or prevent WNV infections in humans, although several vaccine candidates have been proposed [14].

According to the route of transmission, WNV infection may be specifically work-related or have a higher prevalence in some working populations. In fact, considering the low prevalence of cases that are being diagnosed, the true prevalence rate of WNV infection may be underestimated in occupational populations, especially in workers operating in settings characterized by the presence of birds and other animals, and in outdoor workers, in which exposure to infected mosquitos may also depend on geographic location, season and time of duration of outdoor working tasks [15]. In fact, changes in daily work activities because of increased temperatures, with augmented work at dawn and dusk, could correspond to periods when mosquitoes are more active, increasing the risk of disease transmission [16]. Considering that the greater part of WNV infections are subclinical, assessing the occupational prevalence could help not only to protect worker’s health but also provide information on the spread of the virus in the general population.

Hence, the aim of this review is to identify which occupational sectors and populations seem to be more exposed to WNV infection in order to provide evidence for the adoption of targeted policies for the prevention of this vector-borne disease.

## 2. Materials and Methods

### 2.1. Eligibility Criteria

For the aims of this review, only original research publications including cohort studies, case-control studies, cross-sectional studies and case reports were analyzed. In assessing work-related WNV-acquired infection, eligibility criteria included articles that were published in peer-reviewed journals without any chronological restriction until September 2023, in the English language and where WNV infection diagnosis was confirmed using appropriate analytical technique, following the ECDC case definition of WNV infection [17]. Conversely, studies that did not report original results (review, commentaries and letters), generic studies on flaviviruses and zoonotic viruses with non-specific or insufficient information on WNV, studies with no available or reported data on occupational risk for WNV infection and studies on non-human subjects were excluded. Moreover, studies not reporting the diagnostic technique applied for the diagnosis or reporting diagnosis made differently from that proposed by the ECDC definition were also excluded.

### 2.2. Research Question

As a preliminary step, research concepts were defined following the “PICO” (Patient/Population/Problem; Intervention; Control/Comparator; Outcome) strategy as shown in Table 1.

### 2.3. Database and Search Strategy

The present study was designed according to the PRISMA (Prepared Items for Systematic Reviews and Meta-Analysis) guidelines [18]. The protocol for this study was registered in the PROSPERO Database (Registration ID: CRD42022359563).

A comprehensive search was conducted in multiple databases such as PubMed, SCOPUS and Web of Science between January and September 2023. Moreover, a “snowball” approach was applied, with references of the retrieved studies accurately searched for further suitable entries. A combination of the following keywords (“West Nile” OR “West Nile virus” OR “West Nile fever”) AND (“epidemiology” OR “seroprevalence” OR “prevalence” OR “frequency” OR “occurrence”) AND (“occupational” OR “workers” OR “professional”) was searched over these conventional scientific databases (i.e., PubMed, Web of Science and SCOPUS) and modified according to the specific peculiarities of the inquired database. According to the PRISMA Guidelines, articles were initially assessed through title screening for their relevance to the topic. Articles that were positively title- screened were then screened by their abstracts by two investigators (A.S. and E.O). If the content was considered consistent with the design of the present review, the full texts were independently assessed by two investigators (E.O. and A.S.) and abstracted by summarizing

(a) Settings of the study: year, region and targeted groups.
(b) Total number of sampled cases and their demographic characteristics.
(c) Number of reference population (i.e., adults; if available).
(d) Characteristics of the laboratory techniques that were ultimately employed.

In cases of disagreement, a decision was reached following adjudication by a third independent author (P.L.).

We used the Excel tool to extract data from eligible studies. Extracted data were compared, with any discrepancies being resolved through discussion. Eligible outcomes were broadly categorized as follows:

- Diagnosis of previous infection of WNV.
- Occupational exposure to WNV.

The presence of serum IgG and/or IgM anti-WNV antibodies was considered indicative of previous WNV infection, while all activities that could involve both contact with the vector and with infected reservoirs and/or biological fluids were considered occupational exposures associated with contact with the virus.

We collected data on the report (author, year and source of publication), the study (sample characteristics and definition of criteria used for the detection of WNV infection), the participants (clinical features related to the infection) and the research design and features.

### 2.4. Risk of bias Assessment

Risk of bias of collected papers was assessed by means of the National Toxicology Program (NTP)’s Office of Health Assessment and Translation (OHAT) handbook and respective risk of bias (ROB) tool [19,20]. The ROB tool assesses whether the study’s design and conduct have compromised or not the credibility of the link between exposure and outcome, leading to the eventual evaluation of the internal validity of the given study. This assessment was performed through the analysis of six possible domains of bias: participant selection, confounding, attrition/exclusion, detection, selective reporting, and other domains. All of the aforementioned domains were individually rated from “definitely low”, “probably low”, “probably high”, to “definitely high”, while an overall rating for each study was not provided. In fact, the OHAT handbook recommends that even studies with “probably high” or “definitely high” ratings in one or more of the assessed domains should not be removed from consideration of the overall body of evidence. Ratings were provided by two reviewers (E.O. and A.S.); disagreements were resolved by consensus or by input from a third investigator (P.L.) where they did not reach consensus after extensive re-analysis of their ratings.

## 3. Results

**Figure 1:**
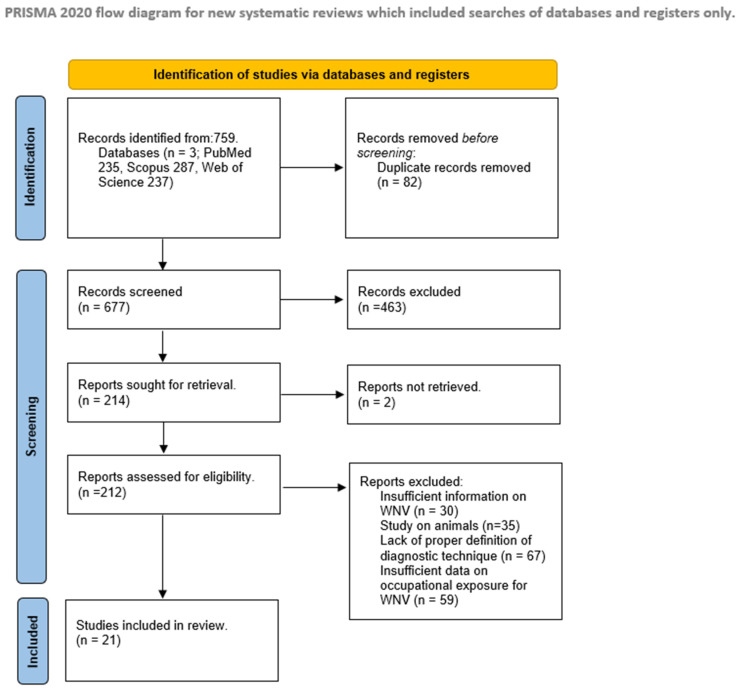
PRISMA 2020 flow diagram.

The systematic search yielded 210 eligible articles with a final selection of 21 studies, seventeen observational studies and four case reports (Figure 1).

### 3.1. Observational Studies

Seventeen observational studies on work-related WNV infection were collected and included in the review (Table 2).

In a 2021 Dutch study conducted by de Bellegarde de Saint Lary [21], the prevalence of WNV infection was evaluated within a group of 157 bird-ringers (high-risk group), after the identification in 2020 in the country of WNV-positive birds and patients exhibiting unexplained neurological disorders. The findings of the study imply that individuals with elevated occupational exposure could serve as a valuable addition to existing surveillance systems for monitoring birds and patients.

The seroprevalence of 19 endemic and emerging viruses, including WNV, was measured in 2020 among healthy military personnel’s participating in a jungle survival course in Manaus (Amazonas state–Brazil) [22]. Among the recruited individuals, 46.6% showed IgG against WNV, suggesting that soldiers can act as a sentinel population to assess the presence of WNV in a specific area.

In the study by Alzuheir et al. (2021) [23], serum samples from 100 veterinarians and 87 horses were collected between August and September 2020 from different cities in Northern Palestine. WNV IgG antibodies showed positive results for 60.9% of horse serum samples and 23.0% of veterinarians’ serum samples, the last not influenced by location, age, experience length and characteristics of working activities.

Given the proximity of the western provinces of Iran to the Middle East endemic area, a cross-sectional study was carried out to assess the presence of WNV in individuals referred to the Blood Transfusion Organization of Kurdistan between 2018 and 2019 [24]. The study gave some information on the occupation of the participants, showing that among the IgG-positive individuals, one was identified as farmer, although occupation and contact with animals was not significantly associated with a seropositive status.

Another WNV seroprevalence study was performed in Eastern Slovakia, on a population of 464 individuals, including 265 patients admitted to the neurology or orthopedic department, and 199 possible high-risk subjects (soldiers, Roma ethnicity, gardeners and agricultural workers). WNV IgM/IgG ELISA analysis showed only three positive cases, one of them suspected to be work-related arising in a shepherdess reporting active gardening and working in a forest [25].

Remoli et al. [26] carried out a seroprevalence survey for WNV in Grosseto province (Tuscany, Italy), where the circulation of arboviruses has been previously documented in animals, to assess the association with occupational exposure. The authors suggested that the limited population sample may be responsible for the lack of WNV cases observed.

The IgM prevalence of nine zoonotic pathogens, including WNV, was investigated in the period 2012–2013 within a pastoral, low-income community adjacent to a wildlife reserve in South Africa [27]. The study involved the recruitment of two groups of participants: those with febrile illnesses and occupationally exposed workers, such as farmers, veterinarians and herders involved in cattle dip-tank activities. Among the recruited subjects, both the two HIA-positive cases in dip-tanksters and the three HIA-positive cases in acute febrile patients were not confirmed by the ELISA test.

In the Enkhtsetseg et al. [28] study, serum samples were gathered from Mongolian military personnel serving as peacekeepers deployed to South Sudan. The research was part of a health screening to monitor serological evidence of exposure to diseases causing febrile illness, including WNV. Out of 632 subjects, 23 exhibited seropositivity for WNV upon return to Mongolia. Notably, among these, 14 individuals displayed IgG against WNV even in pre-deployment sera, indicating prior exposure to the virus.

In a study performed in Greece, occupational information was available for 2897 of 3962 serum samples collected and tested for WNV IgG antibodies by ELISA [29]. All IgG-positive samples were further tested by PRNT and WNV IgM antibodies. WNV IgG antibodies were detected in 58 subjects, with a significant difference among the job groups, although logistic regression did not show any association with occupation and particularly with agricultural working activity.

Similarly, in a seroprevalence survey performed in 2011–2012, 123 veterinarians from South Africa and four from neighboring countries with regular exposure to horses, livestock or wildlife were tested showing 10 WNV-positive samples (7.9%), all from South African veterinarians [30]. The prevalence was not related to age, while it was different according to the geographical area. 

Karakoc et al. [31] performed a seroprevalence survey in nine villages of a Turkish region near the Syrian border, recruiting 182 high-risk workers vs 125 low-risk workers. The WNV prevalence was significantly associated with age higher than 50 years, while no influence of living conditions, repellent use, sleeping outside and using mosquito nets was observed. Multivariate analysis showed a significant influence on the WNV serologic positivity of being in the occupational high-risk group (OR = 2.2, IC 1.02–4.04) and having age higher than 50 years (OR = 5.2, IC 2.76–9.97). 

An epidemiological survey was started in the Veneto region (Italy) in 2008, involving all the 321 workers employed in the farms where WNV-positive horses were identified by veterinary surveillance [32,33]. The observed prevalence was low (1.6%), with two men and three women positive, all asymptomatic, two cases IgM- and IgG-positive and three only IgG-positive, all confirmed by PRNT.

In 2006, a serological survey conducted in the Messina area (Sicily, Italy) involved subjects employed in different occupational settings [34]. Interestingly, the study found no seropositive individuals, suggesting a potential absence of WNV exposure risk in the specific investigated area for recruited workers as well as for the general population represented by blood donors.

In 2002, a seroprevalence investigation was performed after notification of two cases of febrile illness in workers employed at a commercial turkey breeder farm in Wisconsin (US) [35]. Of the 107 total participants, 10 were positive, all of them observed in breeder-farm workers with eight from the first farm. Mosquito exposure and bites were reported as similar between IgM-positive and negative workers. The higher prevalence of WNV IgM in the farm where the first cases were detected suggested the occupational origin of the observed outbreak.

To detect human infections following contact with sick geese, sera were collected from volunteer goose farmers and poultry veterinarians working with sick geese (study group, *n* = 37) and healthy geese (control group, *n* = 39) [36]. A seroprevalence of 89.2% in workers who had close contact with sick geese was detected vs 5.1% in the control group.

Finally, a cohort of male military workers was recruited in a survey conducted to evaluate human prevalence of WNV IgG in Northern Pakistan [37]. Three groups were recruited, including 212 military personnel undergoing training without serologic evidence of acute hepatitis A and B, 192 patients involved in an outbreak of hepatitis E at a military academy and 254 patients admitted to a military hospital for acute febrile illness. The prevalence of WNV IgG ranged from 32.8 to 41.3%, showing an increasing trend with age. PRNT confirmation test was performed only in 15 selected sera from the third group, showing a good sensitivity and specificity of the ELISA test.

**Figure 2:**
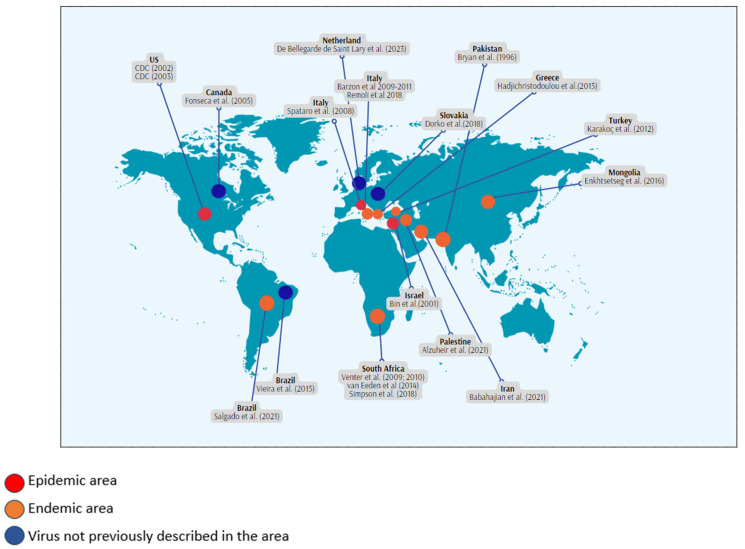
Geographical location and WNV epidemic/endemic status at the time of data collection of the selected studies [21,22,23,24,25,26,27,28,29,30,31,32,33,34,35,36,37].

In Figure 2, the geographic locations of the different studies and the local epidemic/ endemic situation at the time of data collection are reported.

A detailed description of the risk of bias (ROB) assessment on the retrieved studies has been summarized in Table 3.

### 3.2. Case Reports

Four case reports on work-related WNV infection were collected and included in the review (Table 4). In 2014, the first documented case of WNV encephalitis was reported in Brazil, in a 52-year-old ranch worker [38]. The patient reported muscle weakness that arose 2 weeks before the hospitalization during an acute febrile illness, and at the admission to the hospital with acute encephalitis showed flaccid tetraparesis, dysarthria, nuchal rigidity and facial palsy. The patient showed high titers of IgM, while RT-PCR and virus isolation on cerebrospinal fluid (CSF) were negative. Even if the disease did not occur in the context of notified previous equine, avian or human infection or outbreak, serological studies performed among chickens and equines of the patient’s farm showed the presence of specific antibodies against WNV, suggesting a potential undetected diffusion of the pathogen in the area.

An occupational zoonotic human WNV infection was identified in South Africa, in a veterinary student during exposure to infected horse brain while performing an autopsy [39]. The WNV was identified by RT-PCR in the horse brain, and five days after the autopsy the student who removed the brain developed fever, myalgia, and severe headache. Notably, at the time of the incident, the only protective gear worn during the autopsy was gloves. Subsequently, in response to this event, biosafety measures were enhanced, incorporating the use of masks and eye visors to mitigate the risk of similar infections.

Venter et al. [40] also reported a case involving a South African 29-year-old female scientist who had percutaneously inoculated, by a needle-stick injury, cell culture fluid containing lineage 2 WNV strain SPU93/01, isolated from a non-fatal encephalitis human case. Symptoms of backaches, neck stiffness, malaise, rash, mild fever and photophobia spanned 7 to 26 days post-inoculation.

In another work-related WNV infection reported in 2002, a Canadian animal control officer got infected while performing an autopsy on collected sick and dead corvids [41]. The route of infection was the accidental splattering of the infected brain tissues and CSF of Corvus brachyrhynchos onto the officer’s head, eyes, face, neck and right shoulder, and therefore it could be considered the first case of conjunctival transmission. Seven days after exposure, the officer developed symptoms of headaches, dizziness, spiking fevers and sweats, with mild otitis but no neurologic signs. His blood sample collected at this stage was tested for WNV RNA, detected by nucleic acid sequence-based amplification and confirmed by RT-PCR, while his serum sample was negative for IgM antibodies. Two weeks after the initial exposure, a serum sample tested positive for IgM antibodies by ELISA, while the plasma sample became negative for WNV RNA. Moreover, fever, sweat and headache peaked 14 days after exposure, with additional evidence of diminished concentration and impairment of memory, whereas headaches and fatigue persisted up to 8 months after exposure.

CDC reviewed two laboratory-acquired WNV infections in the US in two workers handling WNV-infected samples without other known risk factors [42]. The first case occurred in August 2002 when a microbiologist was performing a necropsy on a blue jay submitted as part of a WNV surveillance program. On the fourth day after the injury, there was the onset of acute symptoms such as headache, myalgia, chills, sweats, dysesthesias, recurring hot flashes, swelling of the host-auricular lymph nodes and anorexia. On the sixth day after the injury, there was a development of macular rash which began on the face and spread to the trunk, arms and legs, but it began to disappear on the ninth day. The serum sample was positive only on the 17th day with WNV-specific IgM and neutralizing antibodies on the 21st day post-injury. The second case, reported in October 2002, was an accidental inoculation in a laboratory worker while trying to harvest the brain of a WNV-infected mouse. The worker experienced illness 3 days after injury which started as upper respiratory infection symptoms and later involved malaise, fatigue, chills and a low-grade fever the following day. Anti-flavivirus IgG antibodies were detected by ELISA in samples collected two days after the onset of fever (day 4).

## 4. Discussion

This systematic review of the literature on the prevalence of work-related WNV suggests that some occupational groups may have a greater risk of exposure to WNV infection, especially in higher epidemic settings, and could represent a sentinel population to monitor the risk of the pathogen’s spread to areas where it was not previously reported.

In recent years, the spread of WNV is increasingly becoming a public health problem, with an estimated economic burden for the management of WNV-related infections in terms of diagnostics and hospitalization amounting to $56.0–$59.9 million per year in the US and recently estimated at 705,107 euros per year in one Italian region alone. [43,44]. Particularly, there is evidence of the circulation of WNV-infected mosquito vectors even in places where no human or animal infection had been reported so far and in previously unreported regions, probably also due to climate change [4]. In fact, the analysis of papers selected for this review showed the presence of WNV cases in European areas previously considered non-endemic, such as the Netherlands, Slovakia and Greece [21,25,29]. In recent decades, Europe has witnessed an increase in the recurrence, expansion of outbreak areas and higher incidence associated with WNV outbreaks, especially in 2010 and 2018 [45]. Particularly, Southern and Central European countries, such as Romania and Italy, have been predominantly affected by WNV outbreaks, with human infections linked to sporadic cases only until the mid-1990s. All these evidences suggest a prospective increase in WNV incidence in the coming years [46].

Among various eco-climatic drivers, climatic anomalies, specifically the temperatures during summer and spring over the last decade, emerged as the predominant factor influencing the recurrent outbreaks of WNV in Europe [46]. These findings hold significance, especially in light of the expected changes in climate that anticipate a rise in the frequency, intensity and duration of extreme weather events like heatwaves, floods, or droughts [47]. These events have the potential not only to enhance the interaction between vectors and hosts, but also between vectors and viruses, favoring the transmission of WNV to humans and leading to an increased occurrence of outbreaks [46]. It is therefore important to consider, in an assessment of work-related risks, how occupational exposure may be affected by the epidemiological context in which the worker is working.

The findings of this systematic review emphasize the existence of a possible increased risk to WNV infection in some occupational settings, although globally the results were not univocal and seem to be strongly influenced by the specific area under study. Specifically, there is the constant threat of WNV exposure posed by jobs involving prolonged outdoor activities in endemic areas, and/or contact with infected animals or with their contaminated body tissues and fluids. In this review, possible higher-risk occupational categories investigated mainly included military workers, veterinarians, outdoor workers such as farmers and agricultural workers, and laboratory personnel.

One of the occupational exposure groups at higher risk of WNV appears to be the military personnel. In particular, according to our results, military personnel carrying out their activities in potentially endemic/epidemic areas have the highest seroprevalence rates. Moreover, military workers, as outdoor workers, should have a higher risk of becoming infected with WNV as they are highly exposed to mosquito vectors. In fact, in contrast to the civilian population, military personnel live in communal settings, undergo training in different environment, and engage in humanitarian aid efforts in challenging conditions. These circumstances, coupled with suboptimal hygiene practices and the stress experienced in the field, elevate the risk of military personnel contracting emerging infectious diseases such as WNV. Consequently, soldiers can serve as a sentinel population and play a crucial role in identifying emerging pathogens [48].

Farmers, agricultural workers, veterinarians and in general jobs in contact with birds and farm animals have also been identified as occupational groups at higher risk for WNV infection. Alzuheir et al. [23] detected a 23.0% WNV seroprevalence among veterinarians in Palestine, which was much higher than that observed in other non-occupational groups from other studies where seropositivity was not associated with either sector of work. Although horses are ‘dead-end’ hosts which do not sustain the circulation of the WNV in nature, veterinarians in the equine specialty have been suggested as the first sentinel of human cases in WNV-infected horses’ areas. In fact, occupational exposure to WNV may be exacerbated by increased exposure risk to mosquito bites following their specific tasks. This agrees with other studies that have long identified veterinarians as having a higher risk of developing zoonotic diseases than other groups of people and professions [30,31,32,33,34,35,36,37,38,39].

In addition, WNV has also been isolated from ticks, although their vector competence is not fully characterized and the knowledge on this possible route of transmission is poor. WNV infection has been confirmed experimentally in both ixodid and argasid ticks, where the latter species maintained the virus in vivo for more than 3 months and was able to infect mice [48], suggesting soft ticks as a potential reservoir for WNV. This may also be another less investigated and not fully elucidated route of occupational transmission.

Finally, as pointed out in the study by Bin et al. [36] and suggested by a case report [41], contact with WNV-infected birds or animals is another occupational risk factor, also supported by the possibility of transmission via aerosols which has been previously demonstrated experimentally [12]. In this case, the risk could be mediated not only by increased contact with the mosquito vector but also by direct exposure to the sick animals that can potentially transmit the virus via aerosols as well as through contact with potentially infected fluids. While viremia in horses and humans is generally too low to facilitate mosquito-borne infection, the viral load in tissues from a fatal case could be sufficiently high. In the case report by Venter et al. [39], for example, the invasive nature of the autopsy may elevate the risk of infection through mucous membranes. To assess the other possible route of transmission of WNV to humans should be one of the major topics of research on this virus, essential for occupational medicine to adopt the highest protection for workers.

Likewise, occupational exposure risk was reported for personnel working with WNV-infected tissues in laboratory settings. In both cases of laboratory-acquired WNV infection included in this review, the most reported means of transmission was accidental percutaneous inoculation, whereas in one case conjunctival transmission was hypothesized. However, in both cases, it is necessary to consider how the epidemic context in which the workers were working may have had an influence, and it is necessary to emphasize in the current risk assessment how laboratory practices may not be comparable nowadays to those indicated at the time of the studies included in the review.

Some case reports and studies, particularly involving bird ringers and turkey farm workers, clearly indicate the central role of birds in the sustenance of WNV as amplification hosts. The latter findings may suggest WNV amplification other than the established putative wild birds. Moreover, it should be investigated if WNV host expansion could interest animals other than birds, as this is not completely unexpected considering that WNV infection has been observed in previously unknown animals such as reptiles.

The analysis of the risk of bias shows that the quality of the selected studies is generally satisfactory. No studies showed a risk of bias high in any of the considered domains, and for seven of the reported studies, the risk of bias was low in all the investigated items. (Table 3). Moreover, the study by Dorko et al. [25] and Alzuheir et al. [23] showed a probably high risk of bias in more than two of the domains. Overall, however, the studies make it possible to adequately assess the results obtained from the literature review.

Our review has a main limitation because in some recruited studies it is not possible to establish a definite causal link between occupational exposure and infection, but only a high probability of infection at work. In addition, it should be considered that in many cases the probability of becoming infected with WNV is also strongly influenced by the epidemiological context in which the workers perform their duties. A further limitation is that some of the studies reported a small number of potentially occupationally exposed employees for each investigated working sector. However, the findings suggest that mosquito-transmitted diseases, such as WNV, should not only be perceived as a risk for the general population and matters of public health concern. Rather, they should be recognized as specific occupational risks that require attention within the framework of occupational health and safety legislation and the need for specific measures in workplaces to reduce the risk of infection, as defined also from specific guidelines [49]. Several preventive actions should be implemented at work, especially in the most exposed categories working in epidemic/endemic settings, such as training and information programs on the risk of infection. Furthermore, in many work areas, vector control is one of the measures to prevent or limit WNV outbreaks. Larviciding reduces the number of vector mosquitoes and serves as a method against the spread of infection. However, its feasibility is limited due to resistance and impact on non-target groups. Adulticidal interventions, e.g., aerial spraying of very low-volume insecticides, have proven to be effective and should accompany other vector control measures and public education campaigns aimed at reducing vector breeding sites, especially in workplaces [50].

To date, no human vaccine against WNV has progressed beyond phase 1 or 2 clinical trials [51]. Several factors have hindered the progression of these vaccines, including challenges in the design and implementation of efficacy studies, concerns about vaccine safety, and costs associated with WNV vaccine programs. Therefore, implementing research in this area appears pivotal to improve the protection of the most exposed individuals, such as outdoor workers. Furthermore, considering the potential spread of the infection in the coming years due to climate change, training programs aimed at improving knowledge about WNV in healthcare workers and occupational physicians appear essential in order to improve epidemiological surveillance against this agent.

## 5. Conclusions

The findings of this systematic review emphasizes that some working categories could be at higher risk of WNV infection, such as military workers, veterinarians, agricultural workers, farmers, and laboratory workers with contact with infected fluids or aerosols, although the findings showed a large variability according to the geographical area and to the specific tasks performed. The need for strategic policies and preventive practices, therefore, seems to be vital in the control of human WNV cases, particularly among higher-exposure workers and in high epidemic settings. In this sense, occupationally targeted programs might be adopted for the prevention of work-related WNV infection, by ensuring early detection and effective strategies in the promotion of public health. While there is currently no singular approach to the prevention of WVN in high-risk populations, quantitative elucidation of the probability of exposure through analyses of surveillance data, particularly those obtained in workplaces, could be a crucial step that can guide public health strategies for WNV control.

## Figures and Tables

**Table 1:** PICO worksheet.

Item	Definition
Population of interest	Workers reporting prolonged outdoor activities or contact with WNV-infected vectors or animals or their biological fluids and with a laboratory diagnosis of WNV infection.
Investigated result	Confirmed West Nile virus infection
Controls	Workers, professionals or the general population without a history of prolonged outdoor activity or contact with WNV-infected vectors or animals and without prior laboratory diagnosis of WNV infection
Outcome	Occupational sectors and population more exposed to WNV infection

**Table 2:** Observational studies on work-related West Nile virus (WNV) infection for systematic review.

Study	Country (Year of Analysis)	Study Population	Analytical/Diagnostic Method	Conclusions/Outcomes Seroprevalence
De Bellegarde de Saint Lary et al. (2023) [21]	Netherlands (2021)	157 bird-ringers vs 58 healthcare workers, 96 blood donors, 94 subjects from Dutch general population (control groups).	IgG protein microarray (possible positive) followed by FRNT ^a^ (confirmed positive)	IgG-possible-positive: 21/157 bird-ringers (13.3%), 0/58 healthcare workers (0.0%), 2/96 blood donors (2.1%), 4/94 general population (4.3%); FNRT confirmed positive: 1/21 bird ringers
Salgado et al. (2021) [22]	Brazil (2014–2015)	298 Brazilian army personnel participating in a jungle survival course.	IgG in-house HIA ^b^	IgG-positive: 139/298 (46.6%)
Alzuheir et al. (2021) [23]	Palestine (2020)	100 veterinarians	IgG ELISA ^c^	IgG-positive: 23/100 (23.0%).
Babahajian et al. (2021) [24]	Iran (2018–2019)	259 blood donors classified by job (7 farmers, 11 army personnel).	IgM/IgG ELISA ^c^	-IgG-positive: 14/269 (5.4%), 1/7 farmer (14.3%), 0/11 (0.0%) army personnel; -IgM-positive 3/269 (1.2%), no farmer or army personnel.
Dorko et al. (2018) [25]	Slovakia (not reported)	265 patients and 199 possible high-risk subjects (103 soldiers, 45 Roma ethnicity, 31 gardeners, 20 agricultural workers)	IgM/IgG ELISA ^c^, positive samples confirmed by PRNT ^d^	IgM/IgG-positive PRNT: 3/464 (0.65%), two patients and one with a possible occupational origin (shepherdess).
Remoli et al. (2018) [26]	Italy (2012)	101 agricultural and forestry workers vs 100 employees in public health offices (not exposed workers)	IgG ELISA ^c^ confirmed by PRNT ^d^	IgG-positive PRNT: 0/201 (0.0%).
Simpson et al. (2018) [27]	South Africa (2013)	64 workers employed at cattle dip-tanks (farmers, herders, veterinary) and 74 patients with acute febrile illness.	IgM in-house HIA ^a^ confirmed by IgM in-house ELISA ^c^	IgM-positive ELISA: 0/64 (0.0%) dip tankster, 0/73 (0.0%) febrile patients.
Enkhtsetseg et al. (2016) [28]	Mongolia (2012–2013)	632 Mongolian army personnel deploying to South Sudan	IgG IFA ^e^	IgG-positive: 23/632 (3.6%), 14/632 (2.2%) positive even in sera collected before deployment.
Hadjichristodoulou et al. (2015) [29]	Greece (2013)	2897 individuals grouped by jobs: (A) 147 farmer/worker, (B) 857 employer, (C) 272 freelancer, (D) 455 housewife/unemployed, (E) 811 child/student and (F) 355 retired.	IgG ELISA ^c^, positive samples analyzed for IgM ELISA and confirmed by PRNT ^d^	IgG-positive ELISA: (A) 3/147 (2.0%), (B) 14/857 (1.6%), (C) 1/272 (0.4%), (D) (1.3%) and (F) (4.8%).
van Eeden et al. (2014) [30]	South Africa (2011–2012)	127 veterinarians	HIA ^b^ and neutralization assay tests	Antibodies positive: 10/127 (7.9%);
Karakoç et al. (2012) [31]	Turkey (2009)	182 high-risk workers (farmers, agricultural workers, unemployed, free traders) vs 125 low-risk workers (housewives, teachers, students, priests)	IgG/IgM ELISA ^c^, positive samples tested by IFA ^e^ and confirmed by MNTA ^f^	MNTA-positive: 38/182 (20.9%) high-risk group vs 14/125 (11.2%) low-risk occupation group.
Barzon et al. (2009; 2011) [32,33]	Italy (2008)	321 workers from farms with WNV horse positive cases.	IgG/IgM ELISA ^c^ confirmed by PRNT ^d^	IgG-positive PRNT: 5/321(1.6%), two of them also IgM-positive.
Spataro et al. (2008) [34]	Italy (2006)	600 healthcare workers, 100 hunters, 80 stable workers as jockey and grooms, 100 fowlers, 100 veterinary surgeons and 500 blood donors.	IgG ELISA ^c^	IgG-positive: no positive case in any group (0.0%).
CDC (2003) [35]	US (2002)	70 workers from 6 turkey farms (57 breeders and 13 non-breeders) vs 23 turkey meat processing facilities workers vs 14 neighborhood residents.	IgM ELISA ^c^ confirmed by PRNT ^d^	IgM-positive PRNT: 10/57 (17.5%) in turkey breeder farm workers vs 0.0% in the other groups.
Bin et al. (2001) [36]	Israel (1998–1999)	37 farmers and veterinarians working with sick geese (study group) vs 39 working with healthy geese (control group)	Neutralization assay followed by IgG ELISA ^c^	IgG-positive ELISA: 33/37 (89.2%) study group vs 2/39 (5.1%) control group.
Bryan et al. (1996) [37]	Pakistan (1986–1987)	Three groups of military workers: (A) 212 in training, (B) 192 involved in hepatitis E outbreak at a military academy and (C) 254 admitted to a military hospital for acute febrile illness	IgG ELISA ^c^	IgG-positive in the three groups: (A) 75/212 (35.4%), (B) 63/192 (32.8%) and (C) 105/254 (41.3%)

^a^ FRNT: Focus Reduction Neutralization Test; ^b^ HIA: Hemoagglutination Immune Assay ^c^ ELISA: Enzyme-linked Immunosorbent Assay; ^d^ PRNT: Plaque Reduction Neutralization Test; ^e^ IFA: Indirect Immunofluorescent Assay; ^f^ MNTA: Microneutralization Assay.

**Table 3:** Tabular representation for the Risk of Bias (ROB) assessment according to the National Toxicology Program (NTP)’s Office of Health Assessment and Translation (OHAT) handbook and respective risk of bias (ROB) tool.

Study	RISK OF BIAS					
	D1	D2	D3	D4	D5	D6
De Bellegarde de Saint Lary et al., 2023 [21]	+	+	+	++	-	+
Salgado et al., 2021 [22]	+	+	-	-	+	+
Alzuheir et al., 2021 [23]	-	+	+	+	-	-
Babahajian et al., 2021 [24]	+	+	+	+	-	+
Dorko et al., 2018 [25]	-	-	+	-	-	+
Remoli et al., 2018 [26]	+	+	+	+	++	+
Simpson et al., 2018 [27]	+	+	+	-	-	+
Enkhtsetseg et al., 2016 [28]	+	+	+	-	+	+
Hadjichristodoulou et al., 2015 [29]	+	+	+	+	+	+
van Eeden et al., 2014 [30]	+	-	++	+	+	+
Karakoç et al., 2012 [31]	+	+	+	+	+	+
Barzon et al., 2009 and 2011 [32,33]	+	+	+	+	+	+
Spataro et al., 2008 [34]	+	+	-	-	+	+
CDC, 2003 [35]	+	+	+	+	+	+
Bin et al., 2001 [36]	+	+	++	+	+	+
Bryan et al., 1996 [37]	+	+	+	+	+	+

Note: D1: possibility of selection bias; D2: exposure assessment; D3: outcome assessment; D4: confounding factors; D5: reporting bias; D6: other bias. ++ : Definitively low; + : Probably low; - : Probably high.

**Table 4:** Case report studies on work-related West Nile virus (WNV) infection included in the systematic review.

**Study**	**Country (Year)**	**Job**	**Possible Route of Transmission/Source of Infection**	**Diagnostic Analysis**
Vieira et al. (2015) [38]	Brazil (2014)	Ranch worker	Not stated/chickens and horses WNV-positive in the worker’s farm	IgM ELISA ^b^, PRNT and HIA on serum.
Venter et al. (2009, 2010) [39,40]	South Africa (not reported)	Veterinary student performing an autopsy	Possible aerosol transmission/infected horse’s brain and spinal cord	Isolation of WNV from serum, confirmed by PCR
		Researcher	Needle-stick injury (percutaneous inoculation)/infected cell culture fluid	Serum positive samples
Fonseca et al. (2005) [41]	Canada (2003)	Animal control officer	Conjunctival (mucocutaneous transmission)/infected crow brain and cerebrospinal fluid	RT PCR ^a^ blood sample positive after 7 days; IgM ELISA ^b^ after 14 days
CDC (2002) [42]	US (2002)	Laboratory worker performing a necropsy	Percutaneous (wound at thumb)/infected bird brain	IgM-positive since the accident: ELISA ^b^ after 13 and 21 days, neutralizing test after 21 days.
		Laboratory worker harvesting brain	Needle-stick injury (percutaneous inoculation) /infected mouse brain	IgM-positive since the accident: ELISA ^b^ after 10 days.

^a^: ELISA: Enzyme-linked Immunosorbent Assay; ^b^: Reverse transcription polymerase chain reaction.

## Data Availability

The data underlying this article are available upon request from the corresponding author.
